# Supplementary material for: Nitrogen-rich covalent organic frameworks with multiple carbonyls for high-performance sodium batteries
Source: Nat Commun. 2020 Jan 10;11:178. doi: 10.1038/s41467-019-13739-5 (PMC6954217; doi:10.1038/s41467-019-13739-5)
Supplement: Supplementary file 1 — Supplementary Information [file 41467_2019_13739_MOESM1_ESM.pdf]

Supplementary Information

Nitrogen-Rich Covalent Organic Frameworks with Multiple Carbonyls for High-Performance Sodium Batteries

Shi et al.

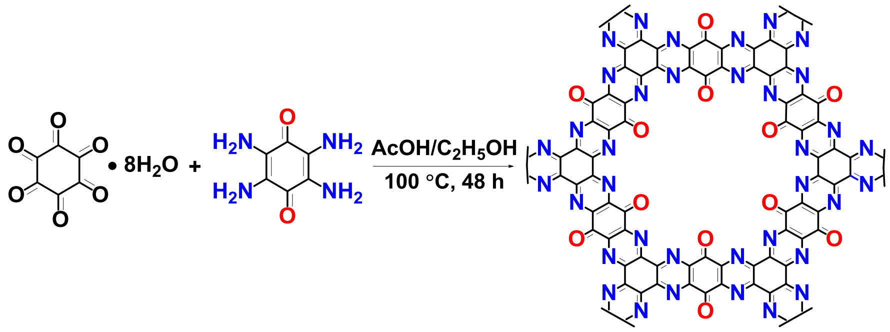

Supplementary Figure 1. Synthesis route and the chemical structure of TQBQ-COF.

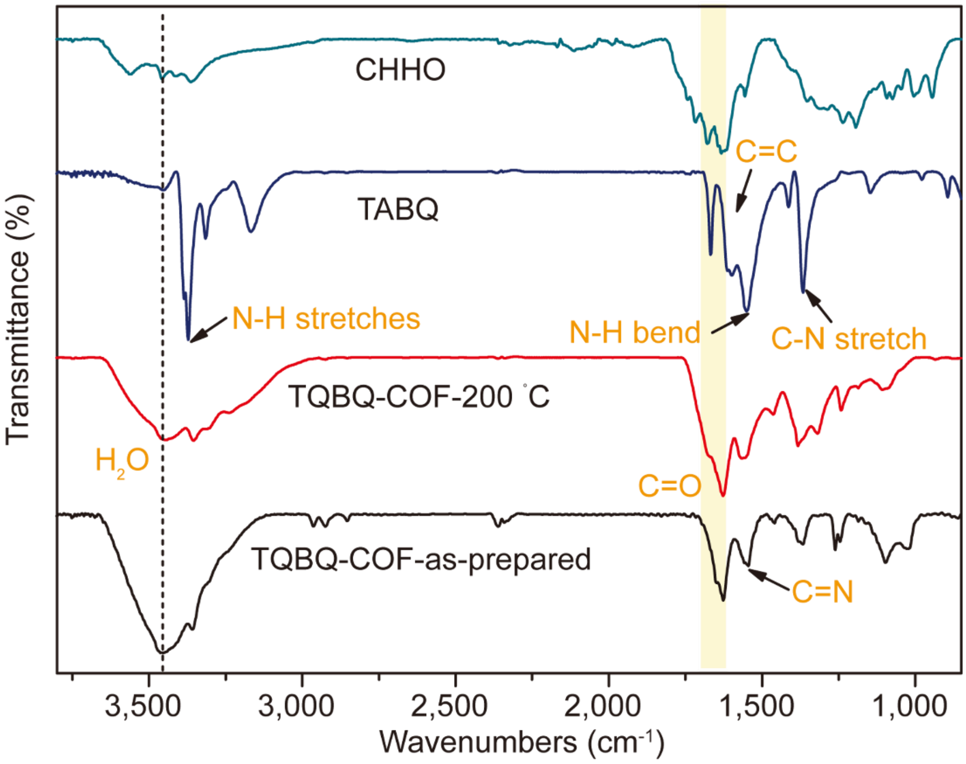

Supplementary Figure 2. FTIR spectra of CHHO, TABQ, TQBQ-COF-200 °C, and TQBQ-COF-as-prepared. The characteristic peaks corresponding to different functional groups are marked above. The peak at 3,373 cm^{-1} can be assigned to the amino groups of TABQ and the peaks ranging from 4,000 to 3,500 cm^{-1} for TQBQ-COF can be assigned to the edge groups (NH_2/OH) and adsorbed substances (such as H_2O, CH_3OH, CO_2) inside the holes. Besides, the strong peak at 1,550 cm^{-1} of TABQ is mostly corresponding to the vibration mode of N-H bend, and the peak at ~1,600 cm^{-1} could be assigned to the vibration mode of C=C.

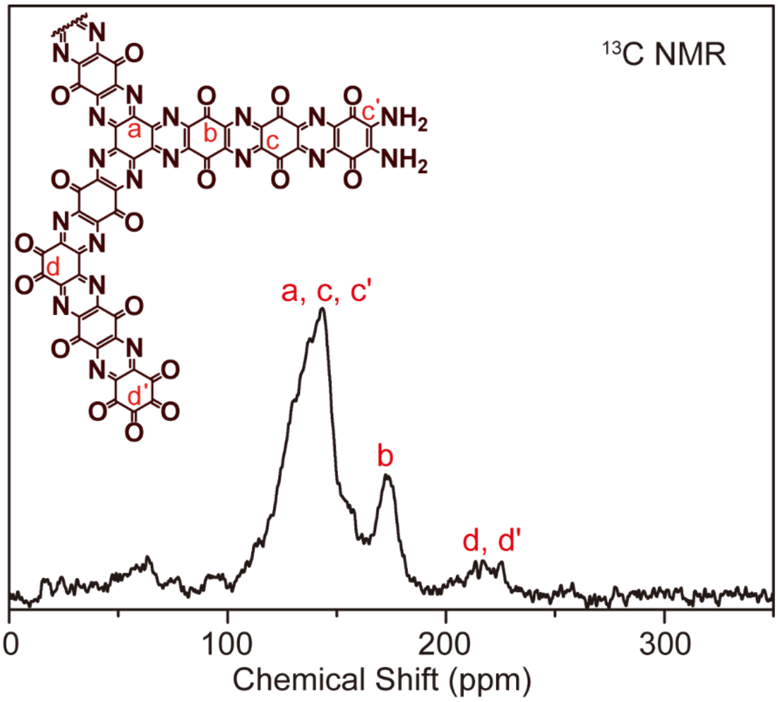

Supplementary Figure 3. Solid-state ^{13}C NMR spectrum of TQBQ-COF (inset: the possible functional groups in the edge of TQBQ-COF).

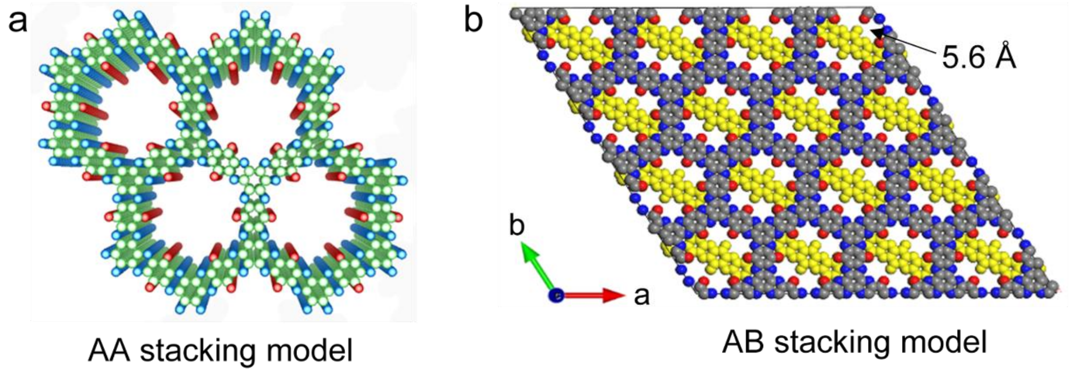

Supplementary Figure 4. Simulated AA/AB stacking models of TQBQ-COF layers. (a) The simulated AA stacking model of TQBQ-COF layers. (b) The simulated AB stacking model of TQBQ-COF layers, with a pore size of ~5.6 Å.

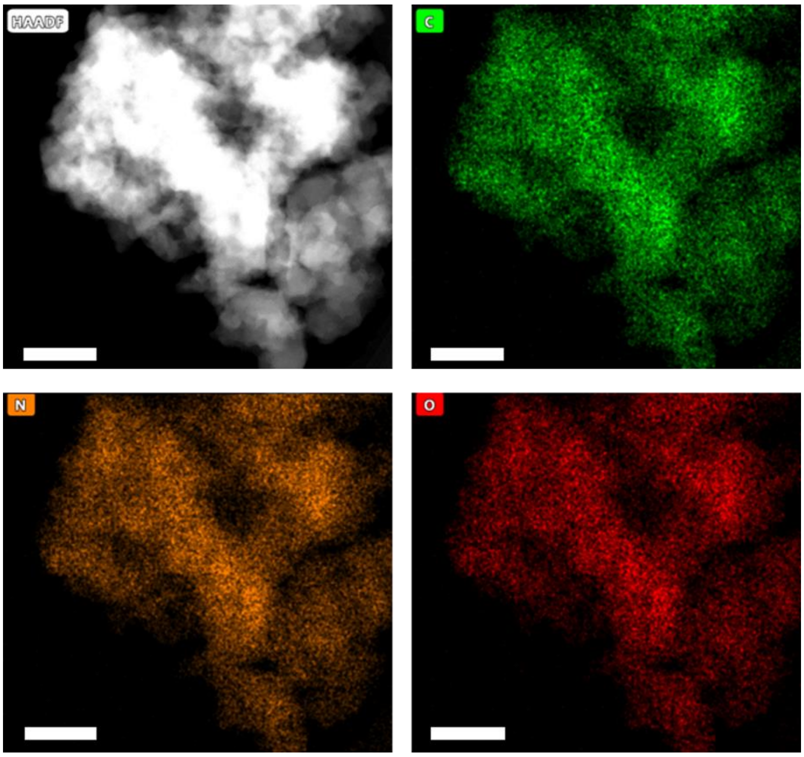

Supplementary Figure 5. Corresponding elemental mapping of C, N, and O elements in TQBQ-COF from the HRTEM image. Scale bar, 200 nm.

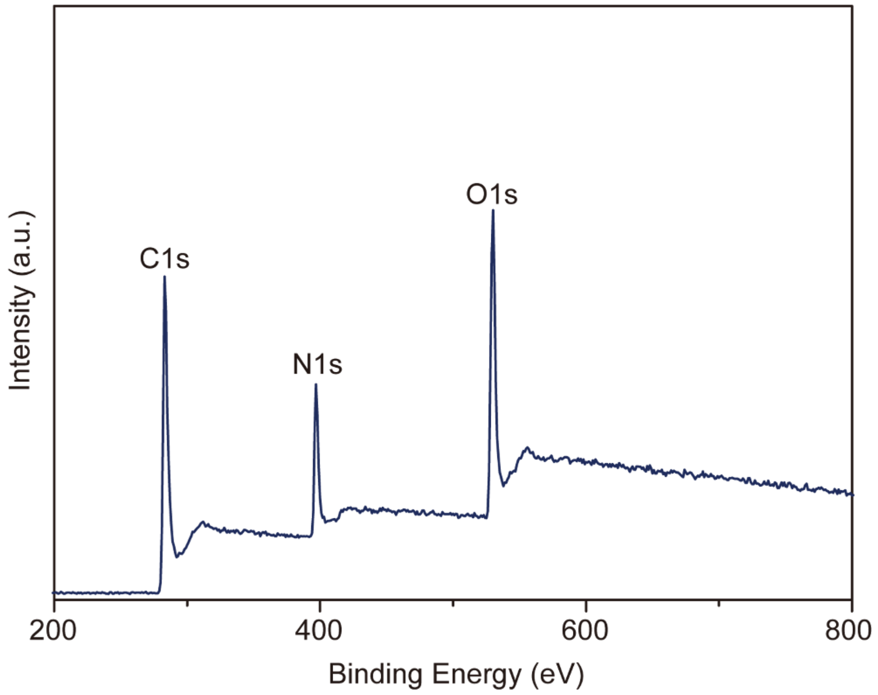

Supplementary Figure 6. XPS survey spectrum of TQBQ-COF material. The nitrogen element accounts for 26 wt%, which indicates a high nitrogen content for the TQBQ-COF material.

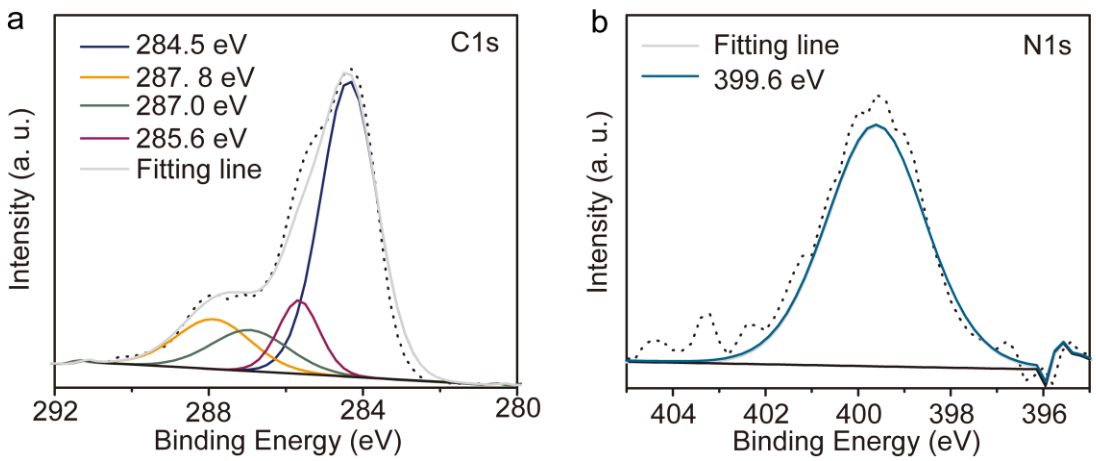

Supplementary Figure 7. High-resolution XPS spectra of TQBQ-COF. (a) High-resolution XPS C1s spectrum of TQBQ-COF. The peak at 285.6 eV can be assigned to the edge groups (C-O/C-N) of TQBQ-COF. (b) High-resolution XPS N1s spectrum of TQBQ-COF. The peak positions of C1s and N1s are corrected with the standard C1s peak (284.5 eV).

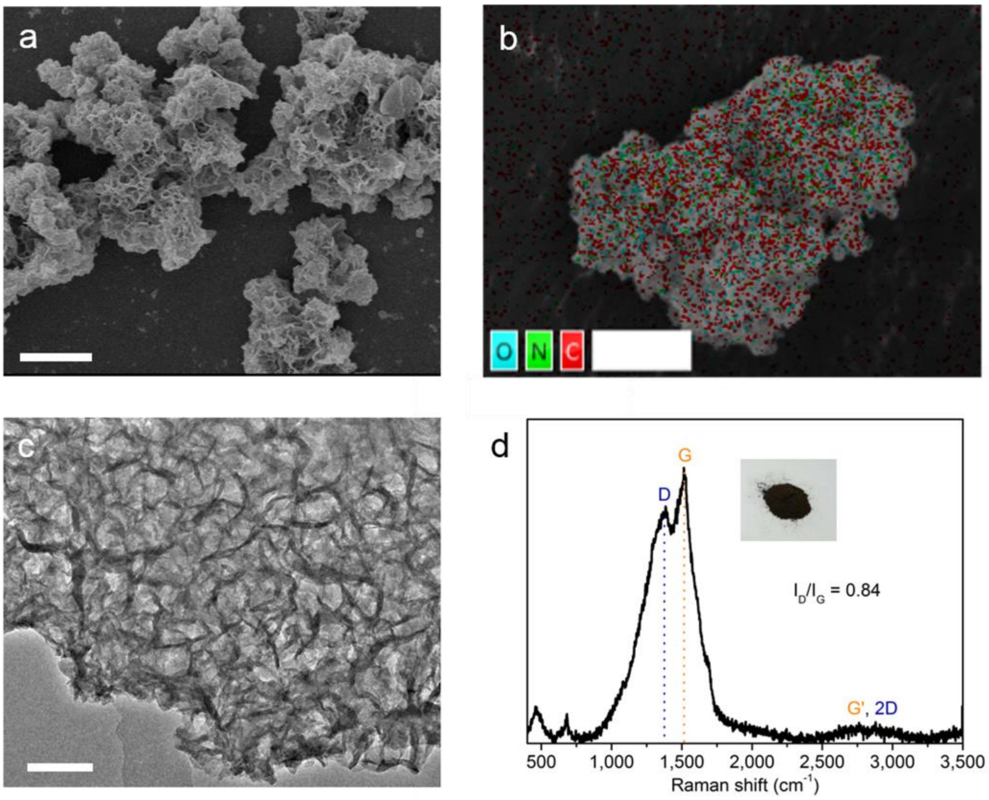

Supplementary Figure 8. Morphology characterizations of TQBQ-COF. (a) SEM image of TQBQ-COF. Scale bar, 1 μm. (b) The corresponding mapping results of TQBQ-COF. Scale bar, 2.5 μm. (c) HRTEM image of TQBQ-COF, showing a porous structure. Scale bar, 100 nm. (d) Raman spectrum of the TQBQ-COF powder. The value of I_D/I_G (0.84) suggests moderate defect level for the 2D structure of TQBQ-COF combined with the AFM and TEM results.

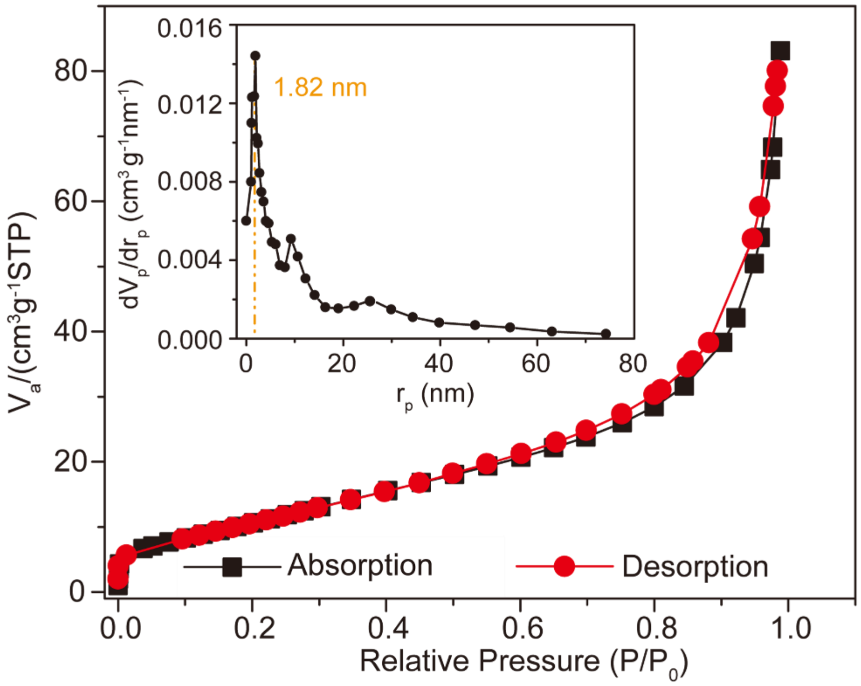

Supplementary Figure 9. N_2 adsorption/desorption isotherms of the as-prepared TQBQ-COF. The inset figure is the corresponding pore size distributions of this sample.

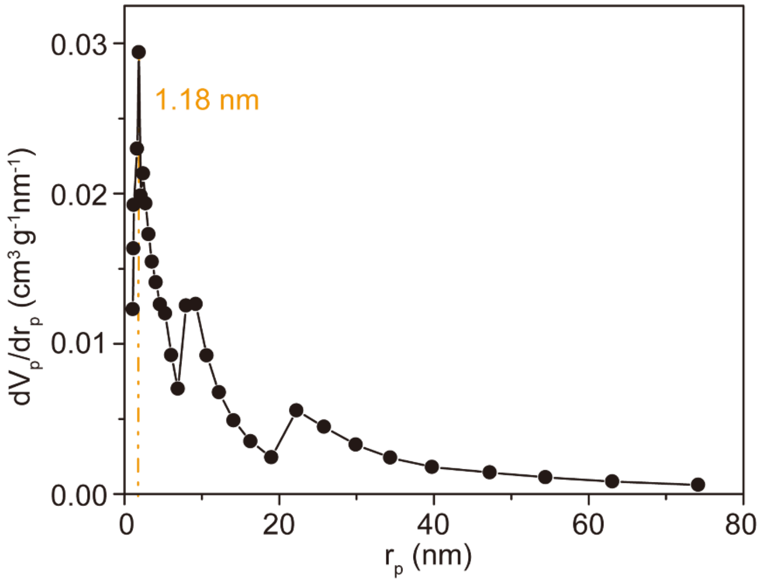

Supplementary Figure 10. The corresponding pore size distributions of the heated sample. The pore diameter is 1.18 nm, which is in agreement with the simulated pore size (11.4 Å) of one TQBQ-COF repetitive unit.

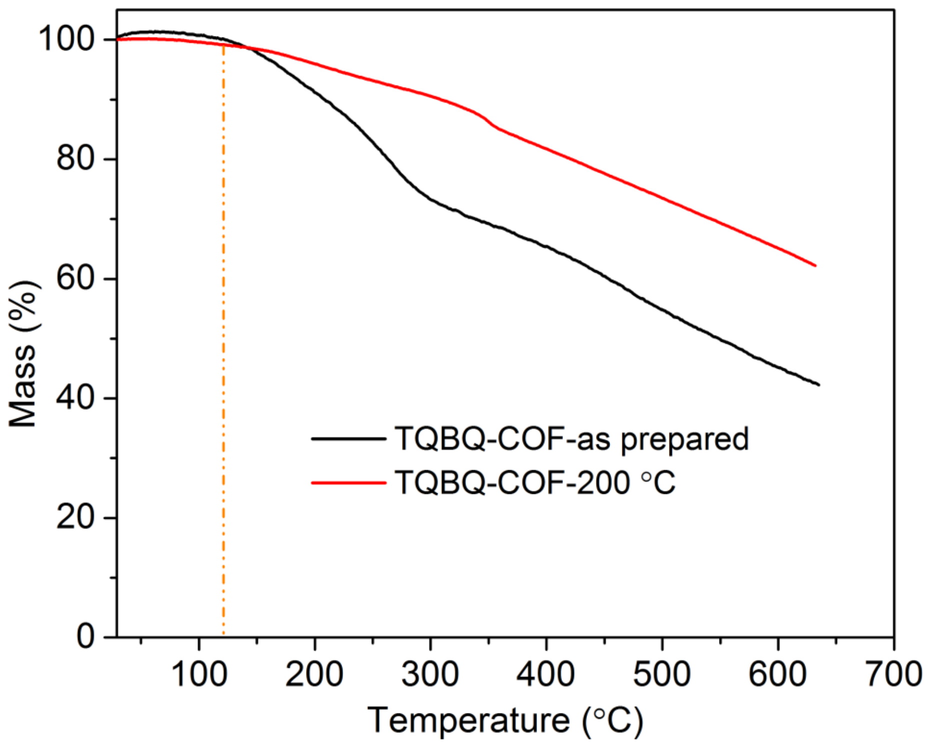

Supplementary Figure 11. TG curves of the as-prepared TQBQ-COF and the heat-treated sample. They are all dried in vacuum before the test, and the remarkable weight loss between 200 and 630 ^{o}C can be ascribed to the decomposition of the TQBQ-COF skeleton.

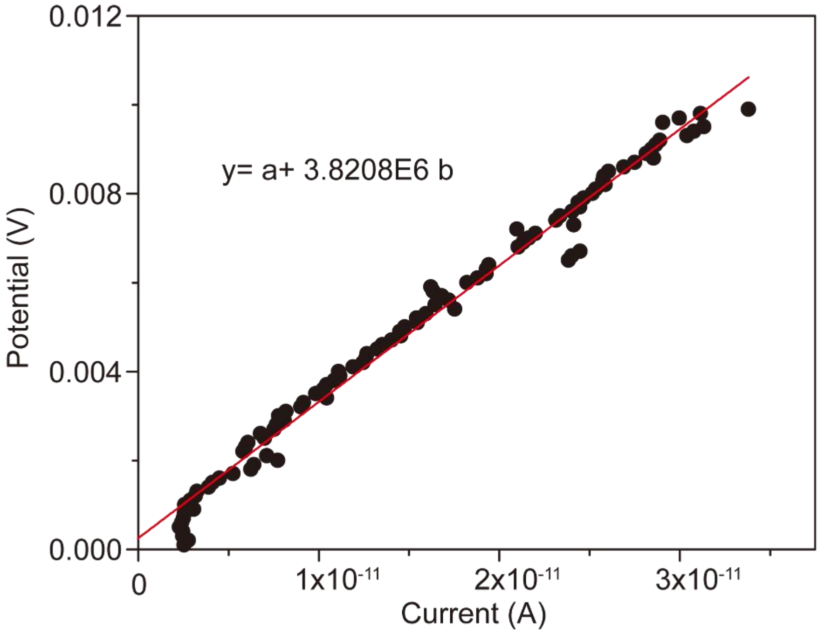

Supplementary Figure 12. Linear sweep voltammetry curve of TQBQ-COF material tested in pellet (d = 13.0 mm, l = 0.1 mm).

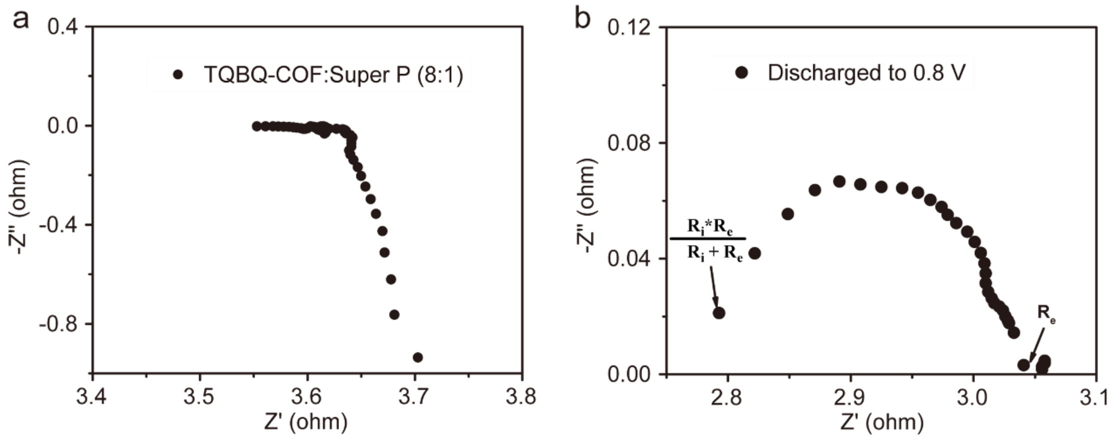

Supplementary Figure 13. The electrochemical impedance frequency response measurement of the pristine and the discharged product. (a) The conductive impedance of the pristine TQBQ-COF/Super P (8:1) mixture. (b) The conductive impedance of the discharged product (0.8 V) of TQBQ-COF/Super P. The thickness and surface area of disk electrode were 0.051 cm and 2.002 cm^2, respectively. Detailed calculation process can be seen in the "Electrical/Ionic conductivity measurements" part of Supplementary Methods.

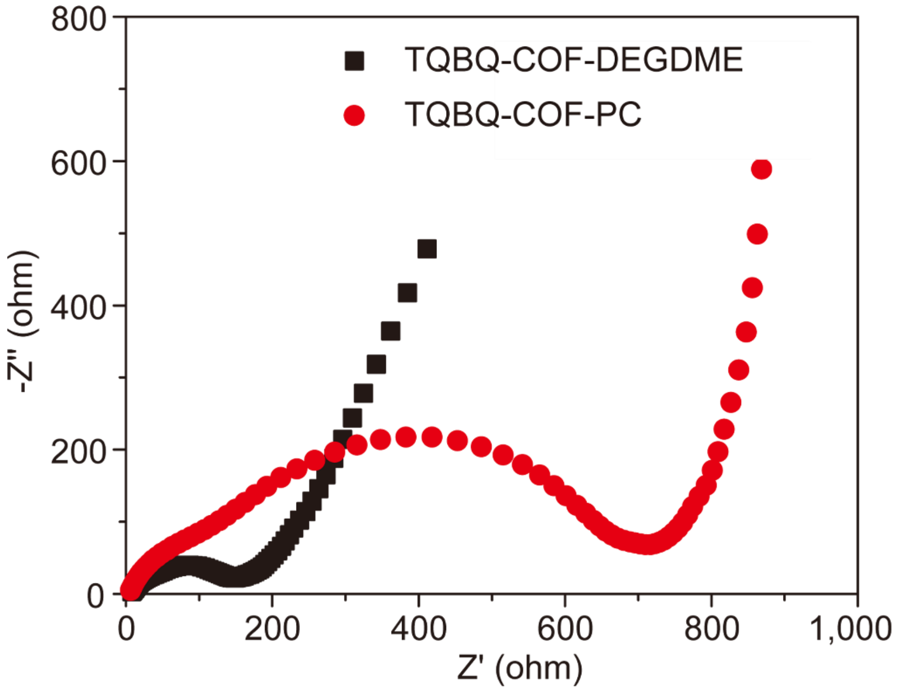

Supplementary Figure 14. The EIS results of the two kinds of electrolytes, 1.0 M NaPF6 in DEGDME and PC, respectively.

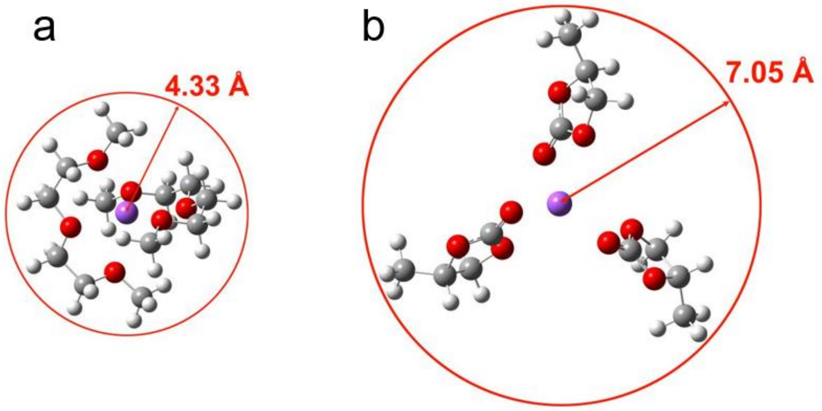

Supplementary Figure 15. The solvation configuration of Na^+ with different electrolytes. (a) The solvation configuration radius of Na^+ in DEGDME-based electrolyte. (b) The solvation configuration radius of Na^+ in PC-based electrolyte. When the coordination number is set as 6 for consistency, the $[NaPC3]^+$ particle (7.05 Å) is much larger in size than that of $[NaG22]^+$ particle (4.33 Å).

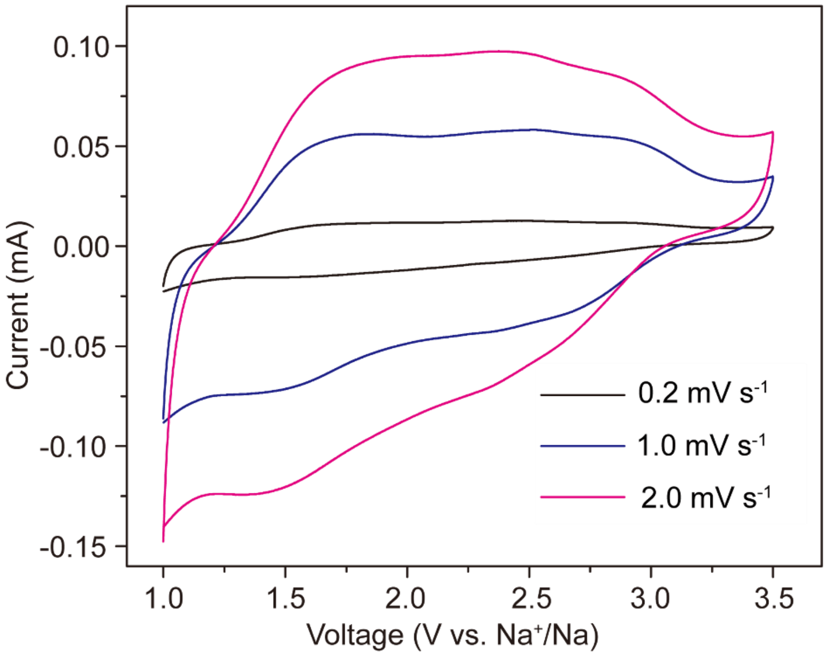

Supplementary Figure 16. CV curves at different scan rates (0.2~2.0 mV s^{-1}) of TQBQ-COF cathode in the PC-based electrolyte. The D_{Na^+} was calculated by the same measurement of TQBQ-COF cathode in the DEGDME-based electrolyte (~10^{-11} cm^2 s^{-1}).

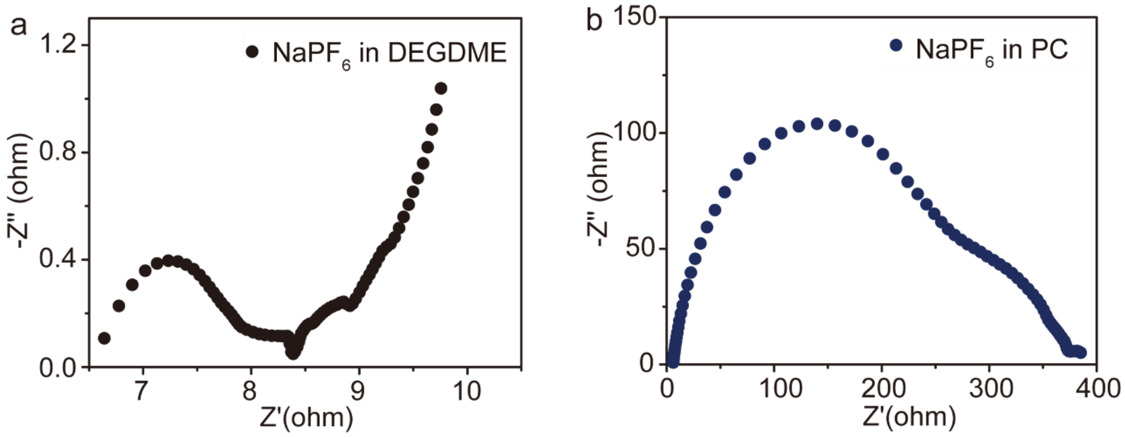

Supplementary Figure 17. The EIS spectra of Na//Na symmetrical cells with different electrolytes. (a) The EIS result in DEGDME-based electrolyte. (b) The result in PC-based electrolyte. The PC-based electrolyte (380 Ω) shows a higher charge transfer resistance than the DEGDME-based electrolyte (8.4 Ω) in the EIS measurement, which shows the interaction between Na anode and PC is the main reason for the high charge transfer resistance in the PC-based electrolyte.

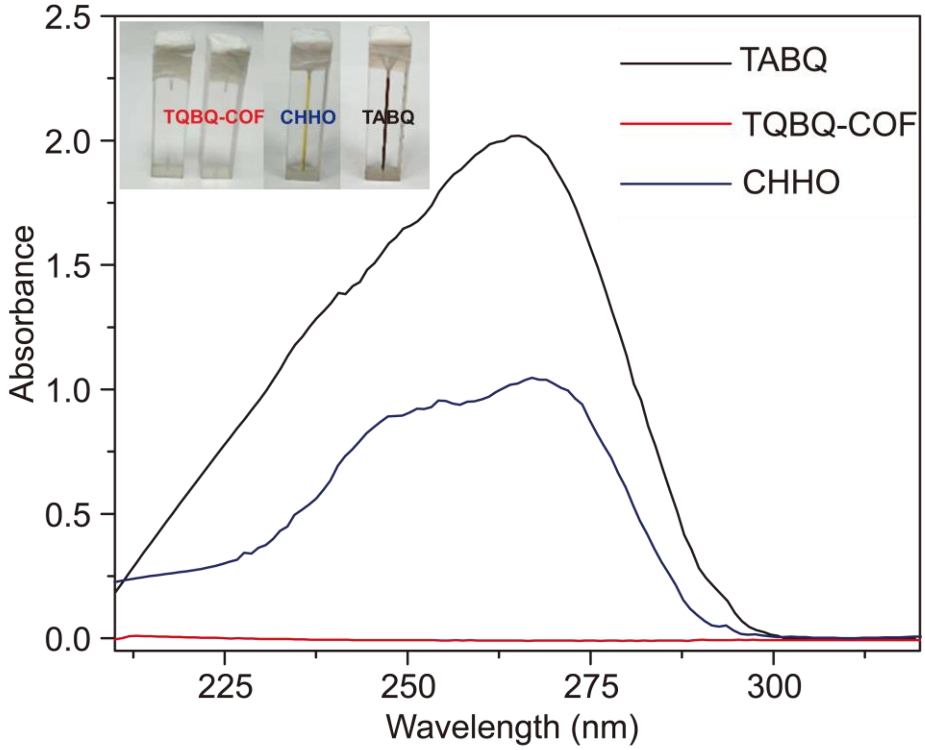

Supplementary Figure 18. UV spectra of 1.0 M $NaPF_6$/DEGDME solutions saturated with TQBQ-COF, CHHO, and TABQ, respectively. The inset figures are corresponding to 1.0 M $NaPF_6$/DEGDME (reference solution), TQBQ-COF-$NaPF_6$/DEGDME, CHHO-$NaPF_6$/DEGDME, and TABQ-$NaPF_6$/ DEGDME, respectively.

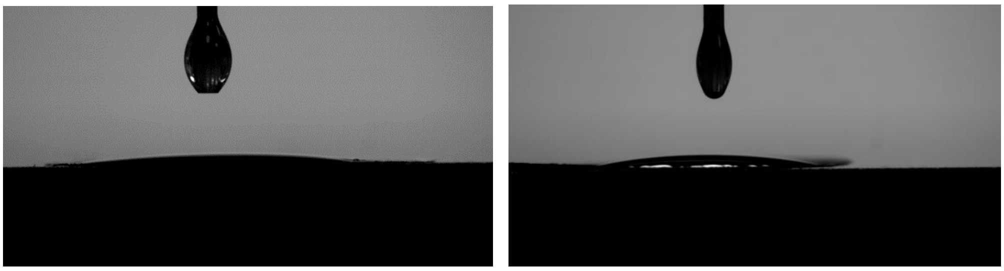

Supplementary Figure 19. Contact angle of the electrolyte of 1.0 M $NaPF_6$/DEGDME with the TQBQ-COF electrode plate. The TQBQ-COF electrode could be wetted in 5 s, showing good compatibility between the electrolyte and the TQBQ-COF cathode.

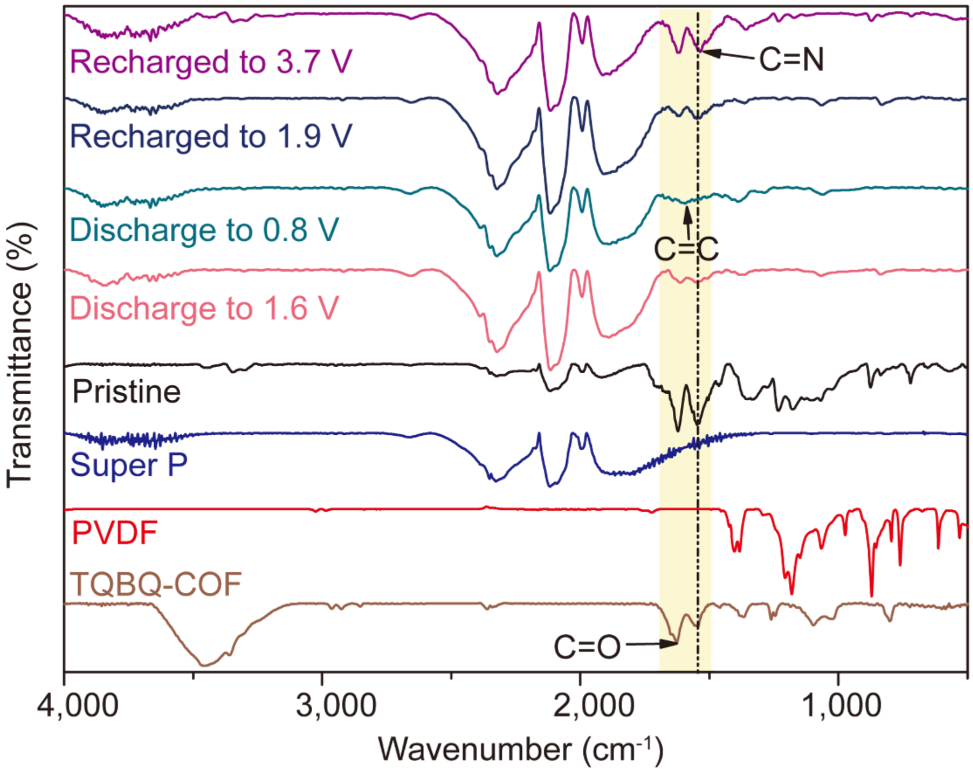

Supplementary Figure 20. FTIR spectra of TQBQ-COF, PVDF, Super P and the products of TQBQ-COF electrodes at different discharged/charged states. The characteristic peaks mainly changed range from 1,700 to 1,200 cm^{-1}.

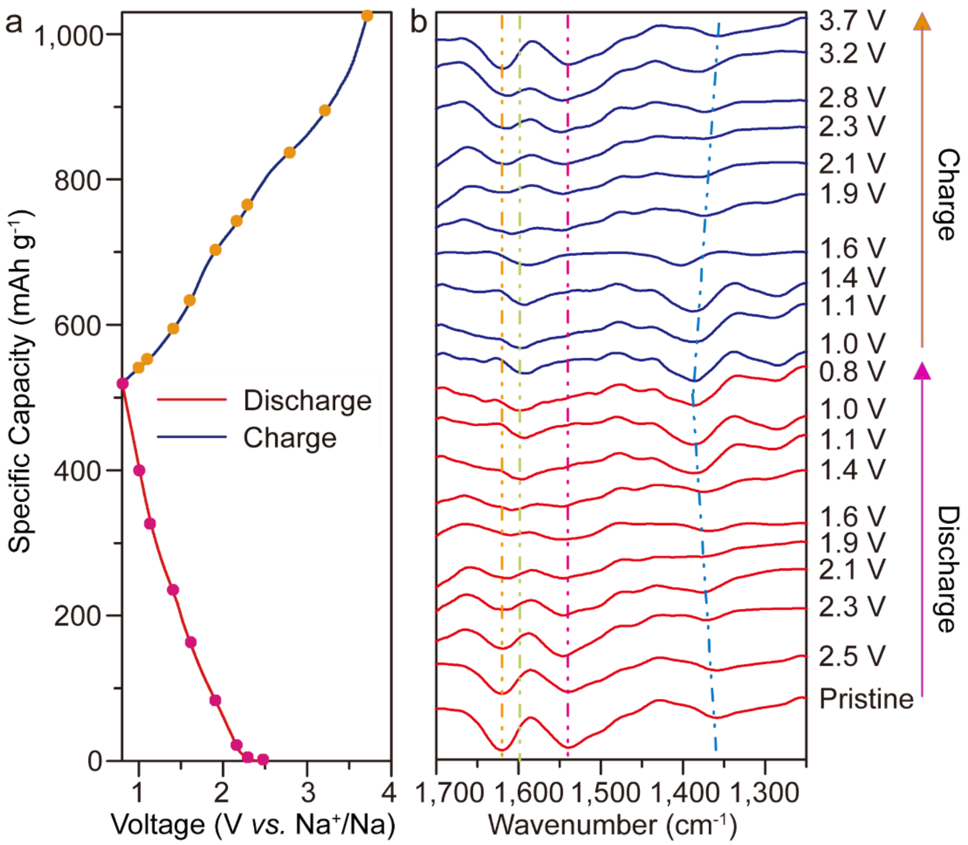

Supplementary Figure 21. Ex-situ FTIR spectra of TQBQ-COF electrodes at different discharge-charge states. (a) The first discharge/charge cycle of TQBQ-COF electrode at 0.02 A g^{-1} in the voltage range of 0.8~3.7 V. (b) Ex-situ FTIR spectra of TQBQ-COF electrodes collected at the indicated states in (a).

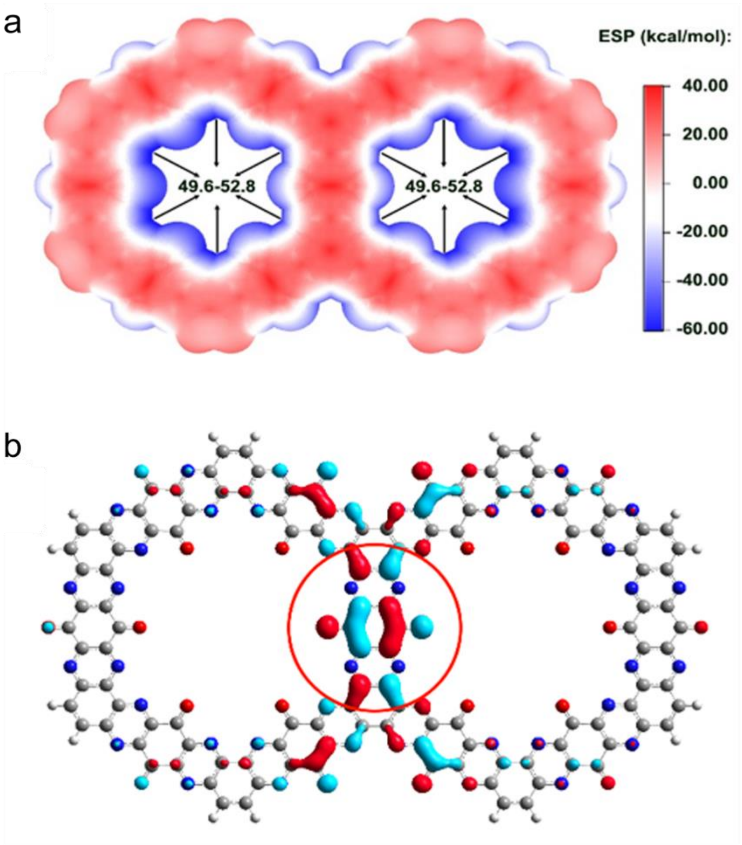

Supplementary Figure 22. The DFT calculations for the two TQBQ-COF repetitive units. The MESP (a) and LUMO (b) patterns of two TQBQ-COF repetitive units. The MESP on the two-unit TQBQ-COF surface minima indicates the same affinity of the para-positioned C=O groups to Na^+ ions, and the symmetrical attribution of LUMO pattern on the C=O groups in the red circle also exhibits the same reaction activity.

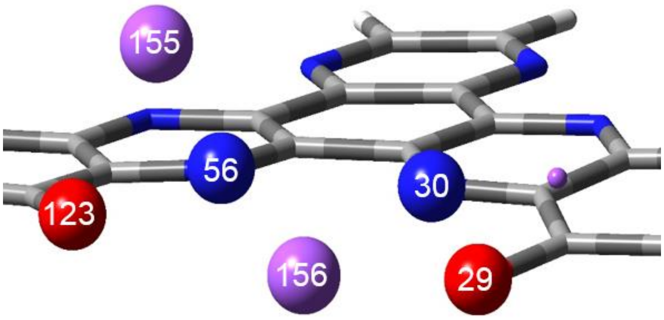

Supplementary Figure 23. The schematic structures of the Na-155 bond and Na-156 bond level analysis in two different coordination environments of TQBQ-COF, respectively.

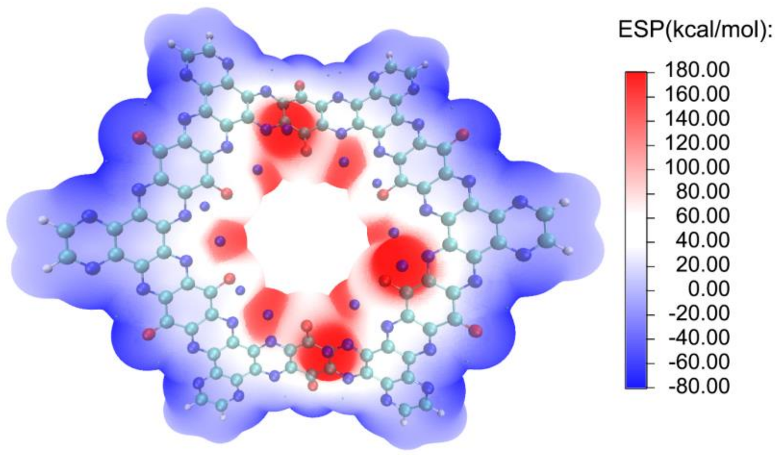

Supplementary Figure 24. Further MESP calculation on Na_{12}TQBQ-COF. There was no Na accommodation site in the unit ring of TQBQ-COF after twelve sodium-ion storage.

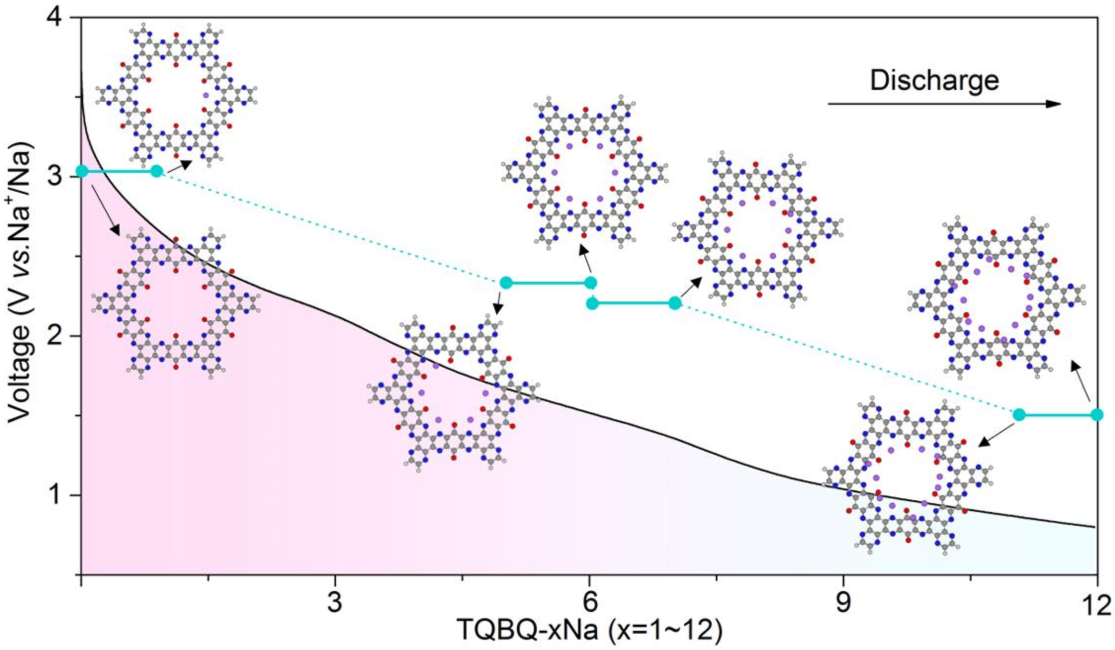

Supplementary Figure 25. The calculated average potentials versus Na/Na$^+$ during the selected sodiation states.

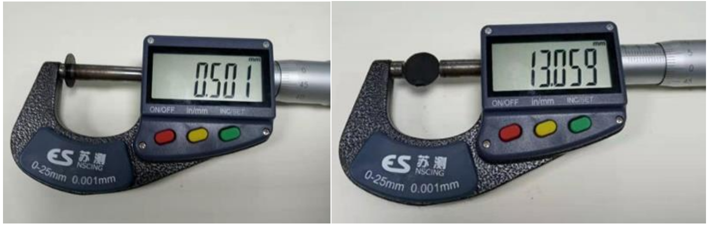

Supplementary Figure 26. The TQBQ-COF pellet was obtained by classic powder's pressing method (14 MPa). The weight, thickness and diameter of the resulting pellet were 109.1 mg, 0.501 mm, and 13.06 mm, respectively. The molar volume (V_m) of the repeating unit of TQBQ-COF is about 383.7 cm^3 mol^{-1}.

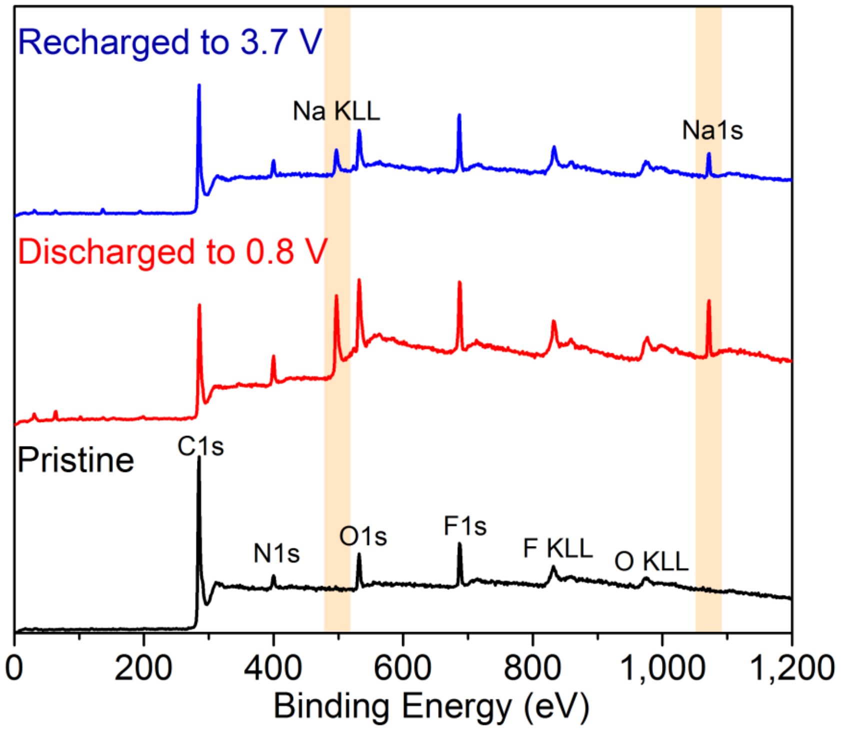

Supplementary Figure 27. Wide XPS spectra of TQBQ-COF electrodes at pristine, discharged (0.8 V), and recharged (3.7 V) states.

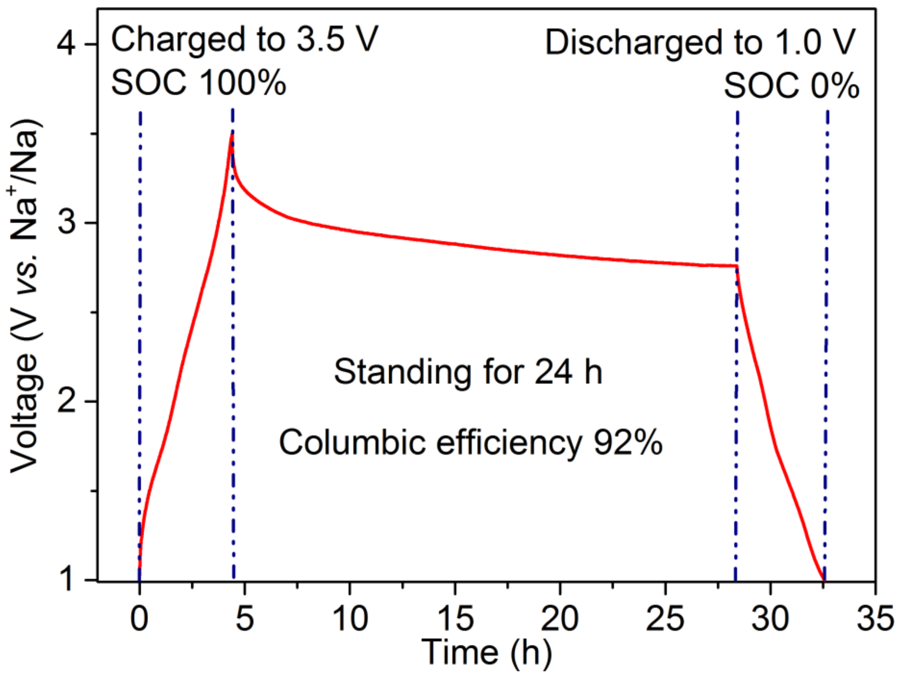

Supplementary Figure 28. Self-discharge performance of the TQBQ-COF sodium battery evaluated by standing for 24 h at 100% state of charging (SOC) after the initial five cycles at 0.2 C (1 C = 400 mAh g^{-1}), followed by discharging to 1.0 V.

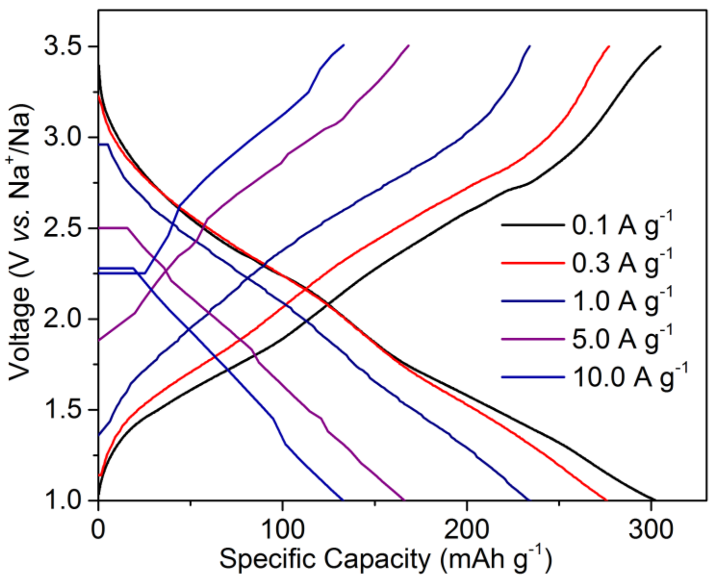

Supplementary Figure 29. Charge–discharge curves of the TQBQ-COF electrode at the current densities from 0.1 to 10 A g^{-1}.

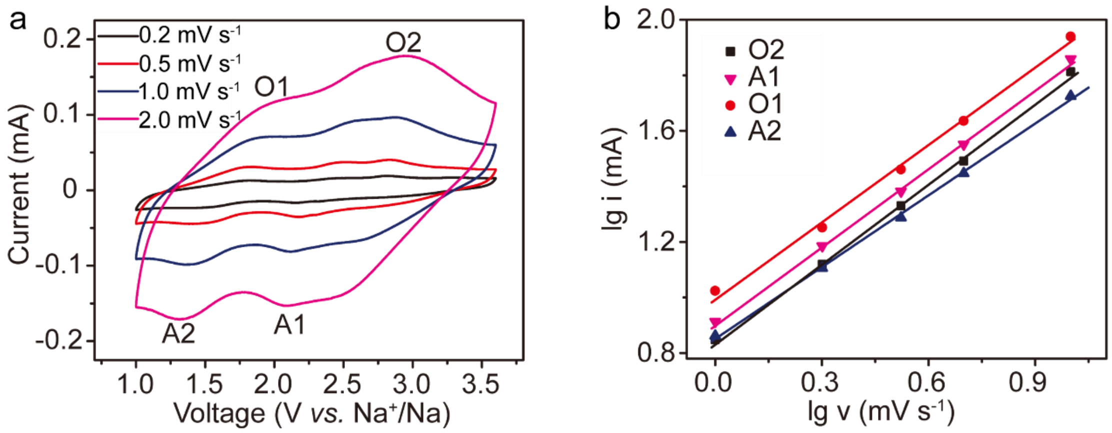

Supplementary Figure 30. CV curves of the TQBQ-COF cathode at different scan rates. (a) CV curves of TQBQ-COF cathode at different scan rates (0.2~2 mV s^{-1}). (b) The corresponding linear fit of the peak current *vs.* the scan rate. The values of b are close to 1.0, showing a capacitive process for Na^+ storage.

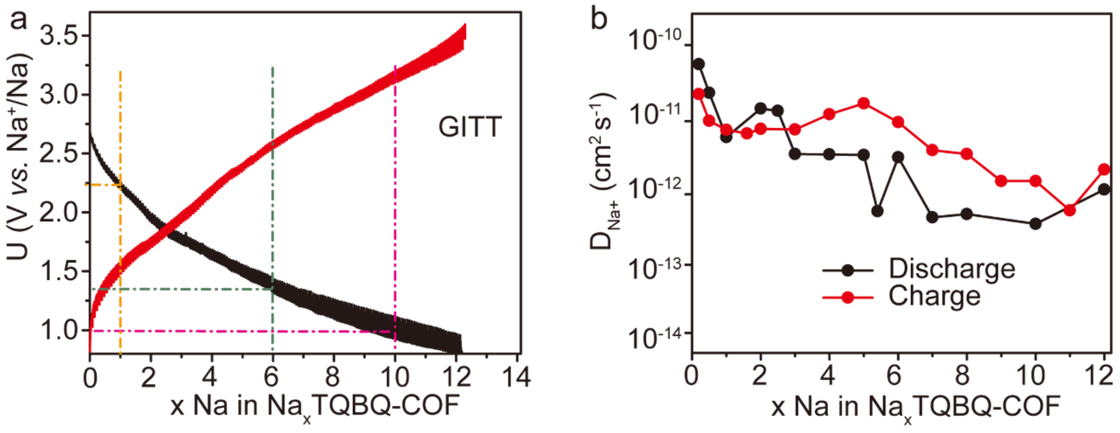

Supplementary Figure 31. The GITT result corresponds to the twelve Na^+ storage in TQBQ-COF. (a) Voltage-composition profile of TQBQ-COF electrode discharged to 0.8 V and recharged to 3.6 V in a GITT mode using an intermittent current rate of C/20 and periodic interruption to reach nearly equilibrium. (b) Diffusivity coefficient of Na^+ calculated from GITT methods versus the sodiation/desodiation of Na_xTQBQ-COF.

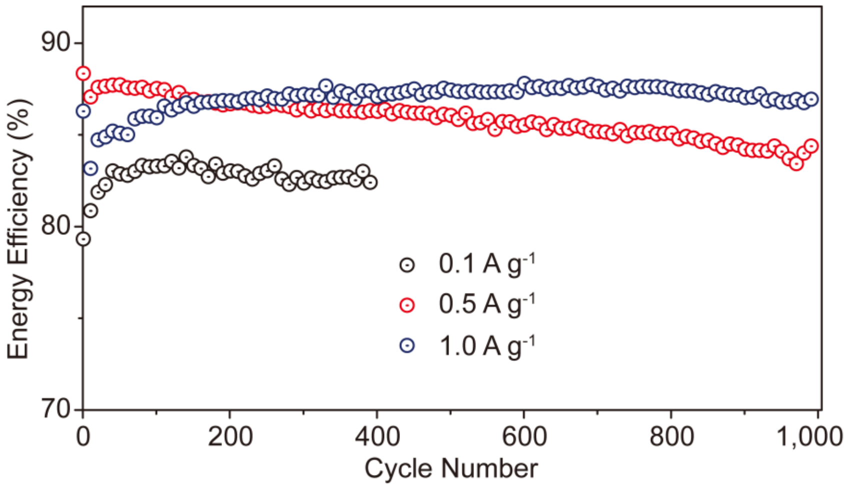

Supplementary Figure 32. The energy efficiency of TQBQ-COF electrodes at 0.1 A g^{-1}, 0.5 A g^{-1}, and 1.0 A g^{-1}, respectively. The values of the energy efficiency are around 85%.

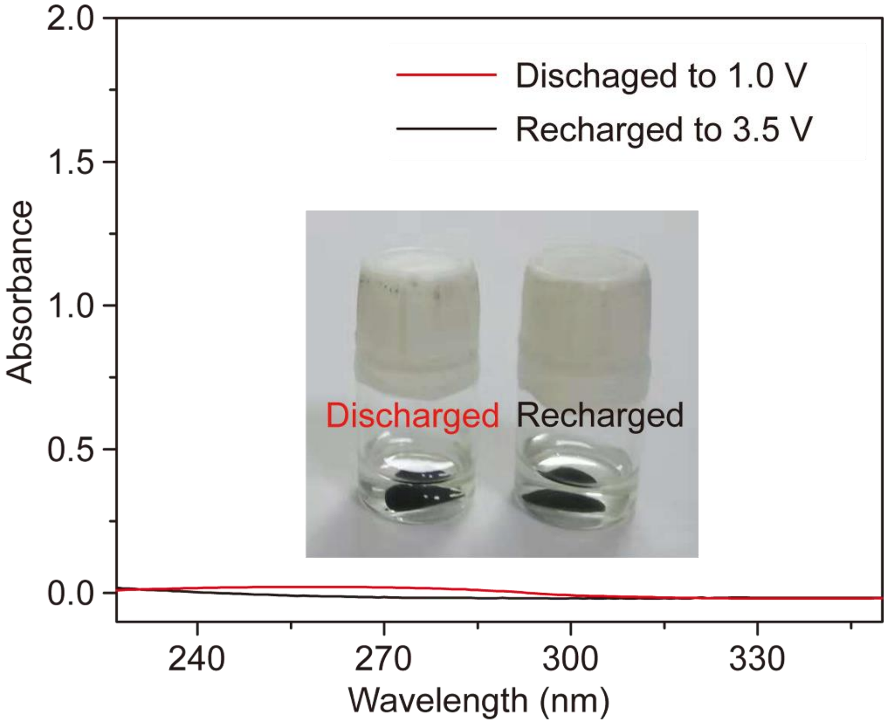

Supplementary Figure 33. UV-vis spectra of TQBQ-COF electrodes in pristine, the discharged (1.0 V), and recharged (3.5 V) states after the immersion in the $NaPF_6$/DEGDME solutions for 24 h, respectively. The insets are the corresponding dissolution behavior of them, showing no color change after discharged and recharged states.

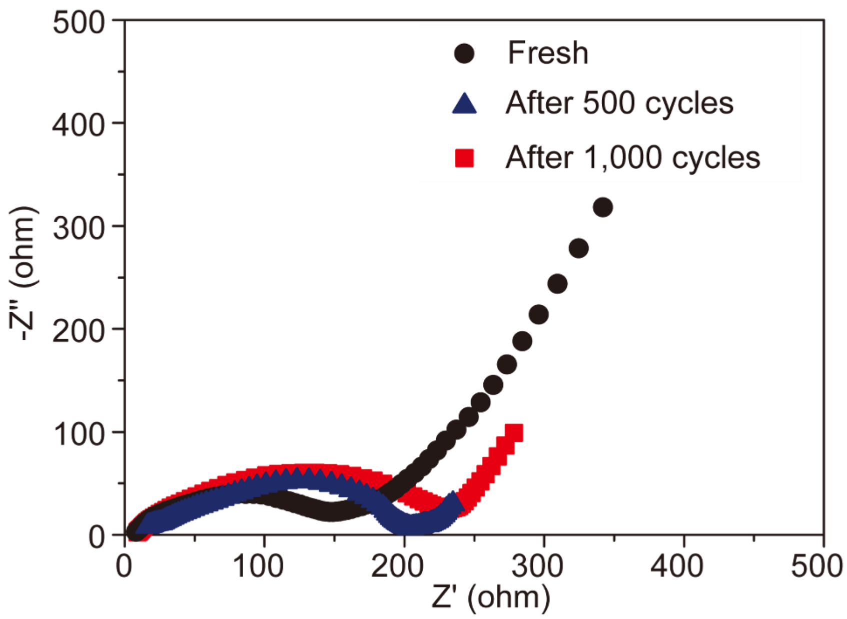

Supplementary Figure 34. The EIS results of TQBQ-COF electrodes in $NaPF_6$/DEGDME electrolyte before and after 500 or 1,000 cycles.

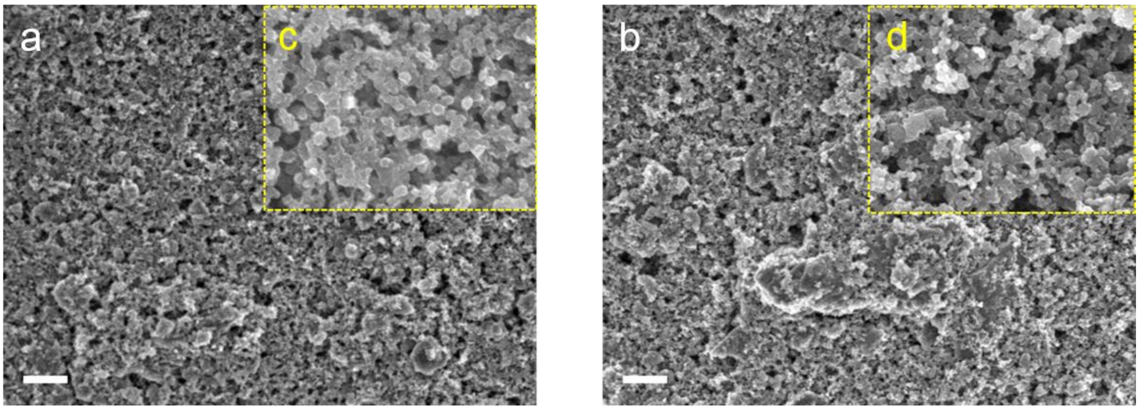

Supplementary Figure 35. Morphology comparison of TQBQ-COF electrodes before and after 500 cycles. (a) SEM image of the pristine state of TQBQ-COF electrode. Scale bar, 2 μm. (b) SEM image of the recharged state of TQBQ-COF electrode after 500 cycles. Scale bar, 2 μm. The insert images (c, d) are the corresponding amplified areas of (a) and (b), respectively, which both show uniform distribution of TQBQ-COF and Super P before and after long cycles.

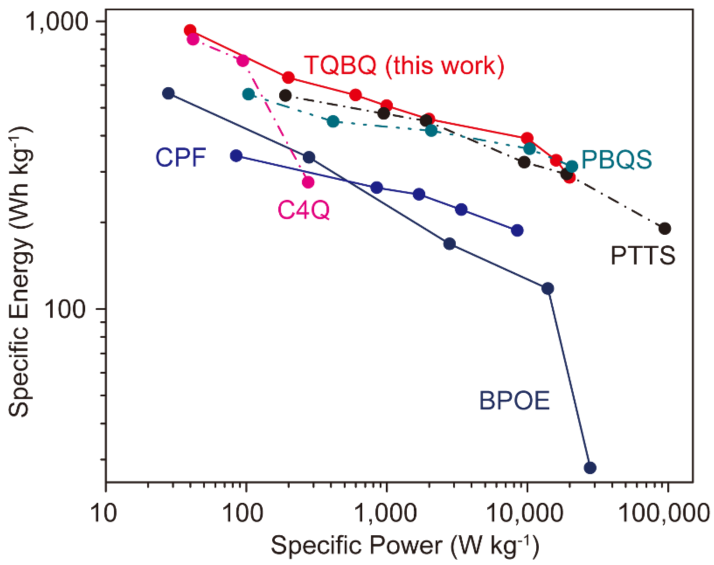

Supplementary Figure 36. Ragone plot of the TQBQ-COF electrode compared with the current reported organic cathodes for sodium batteries (the COFs-based cathodes indicated in solid line).

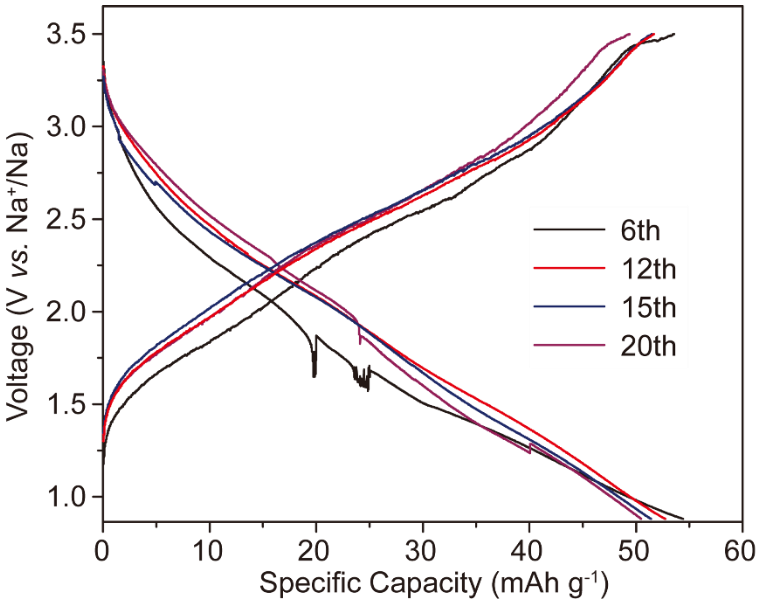

Supplementary Figure 37. The selected charge/discharge curves of pouch-type TQBQ-COF//Na battery at 50 mA. It shows a specific capacity of 54.4 mAh g^{-1} for the 6th cycle based on the whole mass of the pouch-type sodium battery, and the initial five cycles are used to optimize the testing conditions (cell formation, testing press, current density et al.).

Supplementary Table 1. Element compositions of the theoretical calculation, elemental analysis (EA), and EDS mapping results of TQBQ-COF.

	C	N	O	H
**Theoretical (wt. %)**	57.69	26.92	15.38	0
**EA**	53.12	23.76	21.95	2.17
**EDS Mapping**	48.89	21.24	26.43	3.44

Supplementary Table 2. The atomic coordinates of Na_{12}TQBQ-COF.

Center Number	Element	Coordinates(Angstroms)			Center Number	Element	Coordinates(Angstroms)		
		X	Y	Z			X	Y	Z
1	C	-4.519694	-9.372999	-1.881021	79	C	8.042588	5.085396	0.637012
2	C	-3.276814	-8.626956	-1.734586	80	C	6.833824	5.591012	-0.104201
3	C	-3.323088	-7.306217	-1.237345	81	C	5.693785	4.70929	-0.347083
4	C	-4.590126	-6.657772	-0.946964	82	C	5.709502	3.328206	-0.06617
5	C	-5.778859	-7.408551	-0.971795	83	N	11.522903	-0.976892	2.610667
6	C	-5.754536	-8.776082	-1.473922	84	N	11.442597	1.835079	2.432845
7	N	-6.914299	-9.459206	-1.560857	85	O	4.678155	2.507588	-0.431912
8	N	-6.93877	-6.856275	-0.510679	86	O	8.956909	5.816603	0.974615
9	N	-4.552997	-5.351297	-0.606675	87	N	4.558893	5.25483	-0.915154
10	N	-2.216039	-6.612477	-0.95066	88	N	6.862624	6.835028	-0.519803
11	N	-2.079545	-9.183005	-2.040607	89	C	4.58918	6.568932	-1.228931
12	N	-4.480148	-10.626912	-2.371531	90	C	5.752026	7.352638	-1.118803
13	C	-6.9286	-5.605643	-0.115064	91	C	5.743565	8.72551	-1.606951
14	C	-5.737477	-4.763403	-0.211548	92	C	4.527429	9.315287	-2.073703
15	C	-5.752753	-3.375634	0.02246	93	C	3.286444	8.555663	-1.996781
16	C	-6.993466	-2.806018	0.430233	94	C	3.329532	7.208198	-1.574648
17	C	-8.192551	-3.570482	0.708679	95	N	2.219585	6.49606	-1.350883
18	C	-8.216322	-5.046515	0.436585	96	N	2.091492	9.136113	-2.265247
19	C	-0.980931	-8.505228	-1.762179	97	N	4.500788	10.576763	-2.545168
20	C	-1.016185	-7.207203	-1.120087	98	N	6.898339	9.421919	-1.614595
21	C	0.337847	-9.13671	-2.09196	99	C	1.023386	7.107819	-1.474833
22	C	1.524369	-8.469634	-1.44687	100	C	0.990804	8.450552	-2.015807
23	C	1.375887	-7.177713	-0.780034	101	C	-0.327445	9.129153	-2.244847
24	C	0.132281	-6.533032	-0.613163	102	C	-1.486513	8.469087	-1.54429
25	O	0.012808	-5.378905	0.111369	103	C	-1.350045	7.106306	-1.03281
26	O	0.442921	-10.117616	-2.80653	104	C	-0.125224	6.411299	-0.995524
27	O	-9.197519	-5.740445	0.636108	105	O	-0.008068	5.19522	-0.384921
28	O	-4.650952	-2.595115	-0.185127	106	O	-0.449183	10.140533	-2.911959
29	N	-9.286197	-3.01124	1.195672	107	N	-2.463199	6.506868	-0.479628
30	N	-7.035799	-1.467883	0.586412	108	N	-2.575711	9.18162	-1.381261
31	N	2.664674	-9.116339	-1.485441	109	C	-3.641539	8.609557	-0.745543
32	N	2.496718	-6.596517	-0.223932	110	C	-3.578909	7.259378	-0.36195
33	C	-9.285978	-1.674426	1.417586	111	C	-4.843098	9.381551	-0.454732
34	C	-8.151815	-0.912312	1.066778	112	C	-5.964194	8.763071	0.187531
35	C	3.762867	-8.543525	-0.915898	113	C	-5.924378	7.338572	0.492387

36	C	3.664451	-7.269045	-0.32712	114	C	-4.76459	6.604653	0.160931
37	C	-8.129292	0.528315	1.24458	115	N	-4.708322	5.274596	0.27152
38	C	-9.211417	1.186363	1.856812	116	N	-6.973964	6.702165	1.069545
39	C	-10.383759	0.413108	2.250938	117	N	-7.069386	9.46981	0.492131
40	C	-10.429964	-0.999207	2.019825	118	N	-4.87122	10.689586	-0.782369
41	C	5.03869	-9.246994	-0.911082	119	C	-5.801763	4.61435	0.703757
42	C	6.200244	-8.625235	-0.358778	120	C	-6.922604	5.388043	1.199375
43	C	6.111613	-7.266455	0.15809	121	C	-8.085986	4.689497	1.845463
44	C	4.864783	-6.602497	0.151043	122	C	-5.798346	3.197508	0.592888
45	N	5.098605	-10.492019	-1.426667	123	O	-4.796655	2.534118	-0.066781
46	N	7.386006	-9.26431	-0.328521	124	O	-8.960743	5.284513	2.449624
47	N	7.209817	-6.624242	0.622723	125	C	-7.06783	10.755104	0.164596
48	N	4.737967	-5.321371	0.517149	126	C	-5.972391	11.36131	-0.475617
49	C	7.099727	-5.354552	0.963976	127	C	6.845443	10.662025	-2.080959
50	C	5.847875	-4.63196	0.856972	128	C	5.65087	11.237313	-2.549924
51	C	6.276263	-11.099034	-1.387507	129	C	12.571986	-0.220296	2.901349
52	C	7.417651	-10.488479	-0.8366	130	C	12.531624	1.183135	2.816363
53	N	-11.422012	1.053401	2.825357	131	C	-5.634638	-11.274529	-2.451684
54	N	-9.167619	2.536964	2.041766	132	C	-6.847925	-10.692483	-2.042716
55	N	-11.511984	-1.724605	2.362446	133	H	8.372684	-11.010648	-0.812054
56	N	-7.028818	1.173338	0.812896	134	H	6.328336	-12.104147	-1.803113
57	C	-6.949613	2.524319	1.047625	135	H	13.47984	-0.733381	3.214716
58	C	-8.092212	3.192072	1.665907	136	H	13.405554	1.782446	3.066245
59	C	-12.47355	0.316177	3.154741	137	H	7.776284	11.226934	-2.089309
60	C	-12.520343	-1.069766	2.921735	138	H	5.635678	12.25562	-2.934822
61	C	8.328069	-4.65764	1.468934	139	H	-7.958292	11.330568	0.411989
62	C	8.200807	-3.161119	1.562355	140	H	-5.996985	12.417123	-0.740885
63	C	6.916562	-2.513254	1.30597	141	H	-13.312625	0.83175	3.619267
64	C	5.729258	-3.223338	1.029634	142	H	-13.397128	-1.652976	3.198243
65	O	4.525869	-2.581955	0.895026	143	H	-7.782699	-11.246566	-2.112783
66	O	9.347543	-5.252454	1.771508	144	H	-5.608009	-12.287261	-2.850417
67	N	9.278996	-2.484964	1.881588	145	Na	-4.330014	-3.599599	-2.115446
68	N	6.85508	-1.137141	1.366604	146	Na	-2.285923	-4.688282	0.23645
69	C	9.216288	-1.123943	1.903371	147	Na	2.882851	-4.129519	0.00384

70	C	8.008606	-0.471572	1.593034	148	Na	1.155504	-6.42817	1.694565
71	C	10.400668	-0.337126	2.223503	149	Na	5.031781	-1.55513	2.78784
72	C	10.363365	1.087949	2.129265	150	Na	5.230345	0.191471	0.039751
73	C	9.149068	1.746518	1.665762	151	Na	4.693726	3.470608	-2.416547
74	C	7.997631	0.970253	1.405372	152	Na	2.280496	4.489289	-0.29536
75	N	6.884354	1.4994	0.886818	153	Na	-3.006101	4.067596	-0.599487
76	N	9.12391	3.080391	1.436979	154	Na	-1.102501	6.019362	1.350121
77	C	6.86952	2.814716	0.583767	155	Na	-6.471345	1.865771	-1.389184
78	C	8.032509	3.61194	0.917927	156	Na	-5.068392	-0.313051	0.533661

Supplementary Table 3. The Na-155 bond and Na-156 bond level analysis in two different coordination environments of TQBQ-COF.

Bond Order	Na-155	Na-156
O-29	0.00	0.29
O-123	0.35	0.14
N-30	0.00	0.27
N-56	0.22	0.15
…	…	…
Total	0.79	0.87

Supplementary Table 4. The Gibbs free energy values and calculated stepwise voltages versus Na^+/Na during sodiation.

Molecule	Single point energy/hartree	Frequency Correction/hartree	Gibbs Free Energy/hartree	Stepwise Voltage/V	Average Voltage/V
**TQBQ-COF**	-6,082.159613	0.738773	-6,081.42084		
**Na**	-162.2806672	-0.015083	-162.2957502		
**NaTQBQ-COF**	-6,244.564158	0.734607	-6,243.829551	3.073821268	
**Na_5TQBQ-COF**	-6,894.183812	0.735137	-6,893.448675	2.966878707	
**Na_6TQBQ-COF**	-7,056.542842	0.732444	-7,055.810398	1.795204236	2.78942338
**Na_7TQBQ-COF**	-7,218.937479	0.735178	-7,218.202301	2.616460556	
**Na_{11}TQBQ-COF**	-7,868.317285	0.730111	-7,867.587174	1.373304579	
**Na_{12}TQBQ-COF**	-8,030.665117	0.729551	-8,029.935566	1.432459942	1.59035646

Supplementary Table 5. Electrochemical performance comparison of the current reported organic cathodes of sodium batteries.

Active material	Discharge voltage (V *vs.* Na^+/Na)	Reversible capacity (mAh g^{-1}) and rate capability	Capacity retention [%] (Cycle number /Current density)	Ref.
**DSR**	2.0	0.05 A g^{-1}, 484;<br>1.0 A g^{-1}, 371	90.6%<br>(50/0.05 A g^{-1})	**1**
**C4Q**	2.2	0.045 A g^{-1}, 343;<br>0.22 A g^{-1}, 230	78%<br>(900/0.22 A g^{-1})	**2**
**PAQS**	1.8	0.02 A g^{-1}, 233;<br>6.4 A g^{-1}, 165	85%<br>(500/1.6 A g^{-1})	**3**
**PBQS**	2.08	0.05 A g^{-1}, 268;<br>0.2 A g^{-1}, 215	86%<br>(100/0.05 A g^{-1})	**4**
**PIAN**	~2.0	0.05 A g^{-1}, 190;<br>1.0 A g^{-1}, 80	86.3%<br>(150/0.05 A g^{-1})	**5**
**PPTS**	1.9	0.1 A g^{-1}, 340;<br>10 A g^{-1}, 155	86%<br>(1,000/1.0 A g^{-1})	**6**
**CPF**	1.7	0.1 A g^{-1}, 240,<br>1.0 A g^{-1}, 168	82%<br>(1,000/1.0 A g^{-1})	**7**
**BPOE**	2.5	0.01 A g^{-1}, 230;<br>5.0 A g^{-1}, 40	80%<br>(40/0.1 A g^{-1})	**8**
**TQBQ-COF**	~2.0	0.02 A g^{-1}, 452.0;<br>10 A g^{-1}, 134.3	96%<br>(1,000/1.0 A g^{-1})	**This work**

Supplementary Table 6. The mass of sodium metal, electrolyte, and TQBQ-COF (active material) in the pouch-type sodium batteries.

Pouch-cell	Mass
Na metal	0.25 g
Electrolyte	4.0 g Ah^{-1}
TQBQ-COF	0.32 g (6.6 mg cm^{-2})

Supplementary Methods

Materials

Tetraminophenone (TABQ) was synthesized according to a previous reported literatures[9]. Cyclohexanehexaone (CHHO, ~99.0%) was bought from Sigma-Aldrich. All chromatographically pure solvents were directly used into the reactions without further purification.

Electrical/Ionic conductivity measurements

The electrical conductivity of TQBQ-COF was achieved by measuring the pressed pellet of TQBQ-COF powder. To ensure well electrical contact between the TQBQ-COF pellet and the testing chips of the CHI660E Electrochemical Workstation, two stainless steel gaskets were used to sandwich the TQBQ-COF pellet tightly. The resistivity can be obtained by the linear sweep voltammetry (LSV) measurement, as the transient current through the pellet was measured as a function of voltage. The slope of the LSV curve for TQBQ-COF pellet was calculated to be about 3.8208×10^{6} V A^{-1}. The electrical conductivity of the TQBQ-COF pellet was calculated using the following formula:

$$\rho = (V/I)\times(S/L)$$

$$\sigma = 1/\rho \qquad (1)$$

where V is the value of the applied voltage, I is the value of the corresponding current density, S is the area of the TQBQ-COF pellet, L is the thickness of the TQBQ-COF pellet, ρ is the resistivity of the TQBQ-COF pellet, σ is the electrical conductivity of the TQBQ-COF pellet. As L= 0.10 mm, and $S = 3.14\times(0.013/2)^{2} = 1.3267$ m^{2}, so $\sigma = 1.973\times10^{-9}$ S cm^{-1}.

The ionic conductivity of the pristine TQBQ-COF/Super P (8:1) was carried out on the CHI660E Electrochemical Workstation like the above electrical conductivity[10]. The discharged product was the pellet made in the argon-filled glovebox, with the thickness (L) and surface area (S) of 0.035 cm and 2.002 cm^2, respectively. As the R_e = 3.06 Ω, and the value of $\frac{R_i \times Re}{R_i + R_e}$ was 2.79 Ω, the R_i was calculated to be 31.62 Ω. Therefore, from the equation of

$$\sigma_i = \frac{L}{R_i \times S} \quad (2)$$

σ_i was as high as 5.53×10^{-4} S cm^{-1}.

Reaction kinetics of the TQBQ-COF electrodes

To elucidate the reaction kinetics of the TQBQ-COF electrodes, the Na^+ diffusion coefficient (D_{Na^+}) was calculated by the equation[11]:

$$I_p = 2.69\times10^5 n^{3/2} S D^{1/2} v^{1/2} C \quad (3)$$

where I_p is the peak current; n, S, v and C are the electron transference number, the surface area of the electrode, the scan rate, and the concentration of Na^+ in the materials, respectivrly; D is the diffusion coefficient of Na^+. The D_{Na^+} of the TQBQ-COF electrode was calculated to be ~10^{-11} cm^2 s^{-1}. Moreover, the good reaction kinetics of TQBQ-COF electrode can also be verified by the galvanostatic intermittent titration technique (GITT) measurement. The D_{Na^+} of TQBQ-COF electrode was close to 10^{-11} cm^2 s^{-1}. The D_{Na^+} calculated by the GITT method was based on the following equation[12]:

$$D = \frac{4}{\pi\tau}\left(\frac{V_m n_m}{S}\right)^2\left(\frac{\Delta E_S}{\Delta E_t}\right)^2 \quad (4)$$

where τ is the pulse time, n_m is the mole number, V_m is the molar volume, S is the electrode-electrolyte interface area, ΔE_S is the voltage difference between the steady state and the initial state of every step, and ΔE_t is the change of total voltage during a pulse step excluding the IR drop. The molar volume (383.7 cm^3 mol^{-1}) of per TQBQ-COF repetitive unit was calculated by measuring the mass and the total volume of a pressed TQBQ-COF pellet (Supplementary Fig. 26), and the value was acceptable here when compared with some reported organic electrode materials[13].

Pouch-type sodium batteries assembly

The pouch-type cell with a capacity of 81 mAh was constructed with TQBQ-COF cathode, excess Na anode and 1.0 M $NaPF_6$/DEGDME. 0.64 g TQBQ-COF powder, Super P and PVDF (5:4:1 by weight) were mixed by hand grinding. After the addition of NMP solution, the resulting slurry was casted onto the Al foil with the active material loading of 6.6 mg cm^{-2}. The resulted electrodes are dried at 80 ºC for 12 hours under vacuum. To assembly the pouch-cell, Celgard 2400 membrane was used as the separator to lower the whole cell mass, and 1.0 M $NaPF_6$/DEGDME solution was applied as the electrolyte. In addition, the two polytetrafluoroethylene (PTFE) plates (8×10 cm^2) were used to keep the flatness of the electrodes. The mass energy density (*w*, Wh kg^{-1}) was calculated based on the following equation:

$$w = C \times E/m \qquad (5)$$

where *C* (Ah) is the battery discharge capacity, *E* (V) is the average discharge platform, *m* (kg) is a mass of the total pouch sodium battery. As the mass of the full pouch sodium battery (*m*) was 1.49 g (except the mass of the two PTFE plates, which were outside the electrodes), the discharge capacity was 81 mAh, and the average discharge platform (*E*) for the pouch cell was 1.86 V, so the energy density (*w*) was 101.1 Wh kg^{-1}, and the capacity normalized based on the whole mass of the pouch-cell was 54.4 mAh g^{-1}. In addition, the volumetric energy (*w'*, Wh L^{-1}) of the total pouch-cell was calculated to be 78.5 Wh L^{-1}, according to the equation of

$$w' = C \times E/V \qquad (6)$$

where the volume of the pouch-cell (*V*) was 1.92 (6×8×0.04) cm^3.

Supplementary References

1. Lee, M. et al. High-performance sodium-organic battery by realizing four-sodium storage in disodium rhodizonate. *Nat. Energy* **2**, 861-868 (2017).
2. Wang, X. C. et al. Combining quinone cathode and ionic liquid electrolyte for organic sodium-ion batteries. *Chem* **5**, 21 (2018).
3. Deng, W. W. et al. A low cost, all-organic Na-ion battery based on polymeric cathode and anode. *Sci. Rep.* **3**, 2671 (2013).
4. Song, Z. P. et al. Poly(benzoquinonyl sulfide) as a high-energy organic cathode for rechargeable Li and Na batteries. *Adv. Sci.* **2**, 1500124 (2015).
5. Dong, L. et al. Environmentally-friendly aqueous Li (or Na)-ion battery with fast electrode kinetics and super-long life. *Sci. Adv.* **2**, 1501038 (2016).
6. Tang, M. et al. Tailoring π-conjugated systems: from π-π stacking to high-rate-performance organic cathodes. *Chem* **4**, 2600-2614 (2018).
7. Li, H. Y. et al. Large π-conjugated porous frameworks as cathodes for sodium-ion batteries. *J. Phys. Chem. Lett.* **9**, 3205-3211 (2018).
8. Sakaushi, K. et al. Aromatic porous-honeycomb electrodes for a sodium-organic energy storage device. *Nat. Commun.* **4**, 1485 (2013).
9. Manivannan, R., Ciattini, S., Chelazzi, L. & Elango, K. P. Benzoquinone–imidazole hybrids as selective colorimetric sensors for cyanide in aqueous, solid and gas phases. *RSC Adv.* **5**, 87341-87351 (2015).
10. Wang, C. S. & Hong, J. Ionic/Electronic conducting characteristics of $LiFePO_4$ cathode materials. *Electrochem. Solid-State Lett.* **10**, A65-A69 (2007).
11. Levi, M. D. & Aurbach, D. The mechanism of lithium intercalation in graphite film electrodes in aprotic media. Part 1. High resolution slow scan rate cyclic voltammetric studies and modeling. *J. Electroanal. Chem.* **421**, 79-88 (1997).
12. Liang, Y. L., Tao, Z. L. & Chen, J. Organic electrode materials for rechargeable lithium batteries. *Adv. Energy Mater.* **2**, 742-769 (2012).
13. Schon, T. B., Mcallister, B. T., Li, P. F. & Seferos, D. S. The rise of organic electrode materials for energy storage. *Chem. Soc. Rev.* **45**, 6345-6404 (2016).
